# Sources of Resistance to *Fusarium solani* and Associated Genomic Regions in Common Bean Diversity Panels

**DOI:** 10.3389/fgene.2020.00475

**Published:** 2020-06-16

**Authors:** Kimberly Zitnick-Anderson, Atena Oladzadabbasabadi, Shalu Jain, Chryseis Modderman, Juan M. Osorno, Phillip E. McClean, Julie S. Pasche

**Affiliations:** ^1^Department of Microbiology, North Dakota State University, Fargo, ND, United States; ^2^Department of Plant Sciences, North Dakota State University, Fargo, ND, United States; ^3^Department of Pathology and Entomology, Syngenta, Stanton, MN, United States; ^4^Department of Soil, Water, and Climate, University of Minnesota, Morris, Morris, MN, United States; ^5^Department of Plant Pathology, North Dakota State University, Fargo, ND, United States

**Keywords:** *Phaseolus vulgaris*, GWAS, quantitative resistance, Fusarium, root rot

## Abstract

Common bean (*Phaseolus vulgaris* L.) production worldwide is hampered by Fusarium root rot (FRR), which is caused by *Fusarium solani*. Screening for FRR resistance on a large scale is notoriously difficult and often yields inconsistent results due to variability within the environment and pathogen biology. A greenhouse screening assay was developed incorporating multiple isolates of *F. solani* to improve assay reproducibility. The Andean (ADP; *n* = 270) and Middle American (MDP; *n* = 280) Diversity Panels were screened in the greenhouse to identify genetic factors associated with FRR resistance. Forty-seven MDP and 34 ADP lines from multiple market classes were identified as resistant to FRR. Greenhouse phenotyping repeatability was confirmed via five control lines. Genome-wide association mapping using ∼200k SNPs was performed on standard phenotyping score 1–9, as well as binary and polynomial transformation of score data. Sixteen and seven significant genomic regions were identified for ADP and MDP, respectively, using all three classes of phenotypic data. Most candidate genes were associated with plant immune/defense mechanisms. For the ADP population, ortholog of glucan synthase-like enzyme, senescence-associated genes, and NAC domain protein, associated with peak genomic region Pv08:0.04–0.18 Mbp, were the most significant candidate genes. For the MDP population, the peak SNPs Pv07:15.29 Mbp and Pv01:51 Mbp mapped within gene models associated with ethylene response factor 1 and MAC/Perforin domain-containing gene respectively. The research provides a basis for bean improvement through the use of resistant genotypes and genomic regions for more durable root rot resistance.

## Introduction

Fusarium root rot (FRR; caused by *Fusarium solani* [Mart.] Sacc. f. sp. *phaseoli* [Burk.] W.C. Snyder & H.M. Hans) is one of the most prevalent soilborne diseases in bean-growing regions of the United States (Coleman, 2016). Originally described as one species, *F. solani* recently has been recognized as the *Fusarium solani* species complex (FSSC). FSSC is comprised of up to 60 phylogenetically distinct species divided into 10 *formae speciales* (f. sp.) based on host specificity (Matuo and Snyder, 1973). However, previous studies that used the *F. solani* f. sp. designation were pathogenic on other hosts (VanEtten, 1978). Currently, *F. solani* species are characterized by DNA sequences and multilocus haplotypes, rather than the f. sp. classification method, and grouped together into three FSSC clades (O’Donnell, 2000; O’Donnell et al., 2008). Among the multiple species that cause root rot on common bean, *F. solani* has been documented as the most damaging root rot pathogen (Coleman, 2016). Symptoms of FRR on common bean manifest as dark brown to rust colored sunken lesions where lateral roots begin to rot (Abawi, 1989). Lesions on the lower hypocotyl coalesce as the disease progresses and results in complete rot of the root system (Abawi, 1989). When left unmitigated, FRR can cause up to 84% yield loss (Schneider et al., 2001).

Managing FRR can be difficult due to the durability and extended viability of chlamydospores in soil and plant debris (Katan, 2017). Current management strategies include the use of seed treatment chemicals, avoiding infested fields, crop rotation, and planting certified seeds. However, the most sustainable and durable approaches for controlling the disease is genetic resistance (Rubiales et al., 2015). While foliar disease resistance is a target for crop improvement, less emphasis has been given to breeding for root rot resistance in common bean and there are fewer sources of root rot resistance available. Although commercial cultivars are known to have limited FRR resistance, multiple studies have characterized and identified sources of resistance within common bean germplasm collections (Román-Avilés and Kelly, 2005; Bilgi et al., 2008; Nicoli et al., 2012; Hagerty et al., 2015; Nakedde et al., 2016; Vasquez-Guzman, 2016).

Common bean is divided into the Middle American and Andean gene pools (Mamidi et al., 2011; Bitocchi et al., 2013; Schmutz et al., 2014). The Middle American gene pool is further divided into four races which include Durango, Jalisco, Mesoamerican, and Guatemala (Singh et al., 1991; Beebe et al., 2001; Blair et al., 2009), while the Andean genepool is comprised of races Nueva Granada, Peru, and Chile (Singh et al., 1991). In North America and the United States, the most frequently cultivated common beans are members of market classes within races Durango and Mesoamerican of the Middle American genepool and Nueva Granada of the Andean genepool (USDBC 2017). The Andean Diversity Panel (ADP; Cichy et al., 2015) and Middle American Diversity Panel (MDP; Moghaddam et al., 2016) reflect modern genetic diversity in two common bean gene pools and were used extensively to study the genetics of abiotic and biotic stresses in common bean (Zuiderveen et al., 2016; Soltani et al., 2017, 2018; Oladzad et al., 2019b). In 2013, 310 ADP genotypes were evaluated in the field in Minnesota for resistance to root rot (Vasquez-Guzman, 2016). The major contributor to disease was *F. solani* f. sp. *phaseoli* and only five genotypes were considered resistant to FRR.

From a genetic perspective, a QTL mapping study of Middle American recombinant inbred lines developed from landrace Puebla 152 and the commercial black cultivar ‘Zorro’ detected one QTL on Pv05 associated with a resistance to FRR (Nakedde et al., 2016). Hagerty et al. (2015), placed a QTL associated with FRR resistance on Pv03 using a snap bean RIL population. Nine QTL explaining 5–53% of the phenotypic variation were identified in two inbred back cross line populations (IBL) developed from crosses between a Mesoamerican black bean with an Andean kidney bean and an Andean cranberry bean (Román-Avilés and Kelly, 2005). Finally, one genome-wide association study (GWAS) used field screening data of the ADP and 3,525 single nucleotide polymorphism (SNPs) and detected a genomic region on Pv04 centered at 3.3 Mbp associated with root rot resistance (Vasquez-Guzman, 2016).

A crucial step in developing resistant varieties is a reproducible protocol to screen for pathogen resistance under controlled conditions. Abiotic factors, including soil moisture content and temperature, can dramatically influence pathogen colonization or root development (Peña et al., 2013), resulting in inconsistent phenotypic evaluations. Improving the phenotypic methods of screening for resistance provides more robust and accurate phenotypic data that increases the power of GWAS to identify and map resistance QTL. GWAS results can vary based on methods either quantitative or qualitative, used to classify phenotypic data (Oladzad et al., 2019b). Therefore, another important aspect of this study was to develop GWAS results from quantitative, three-class, and binary scoring systems.

The objectives of this research were to: (1) develop a reproducible greenhouse evaluation system for FRR in common bean, (2) identify highly resistant genotypes to FRR in the ADP and MDP, and (3) discover genomic regions and potential candidate genes associated with resistance using GWAS. This is the first large scale-study to evaluate the ADP and MDP in the greenhouse and identify genomic regions involved in resistance to FRR.

## Materials and Methods

### Greenhouse Assay Development and Phenotyping

Results from preliminary experiments indicated that very little to no disease symptoms were observed when only one *F. solani* isolate was used for inoculations (data not shown). Therefore, nine isolates of *F. solani* obtained from diseased dry beans in the Red River Valley region were chosen for inoculation. The isolates used were 09/RG/BF212, 08/RG/BF128, 09/RG/BF261, 08/RG/BF199, 09/RG/BF307, 09/RG/BF279, Fs101.5ND15, 08/RG/BF133, and 09.113.03. Isolates were confirmed as *F. solani* via amplification of the translation elongation factor alpha 1 (TEF-1α) with primers EF-1 and EF-2 (Knutsen et al., 2004). Isolates were grown for 1 week on 0.5 × potato dextrose agar (Difco^TM^ Potato Dextrose Media, BD) in 60 × 15 mm plates. Macroconidial spore suspensions were prepared for each isolate. Under sterile conditions, agar containing fungal growth from one 100 mm Petri plate from each isolate was cut into approximately 1 cm square pieces and added to a 2 L Erlenmeyer flask containing 1 L of CarboxyMethly-Cellulose broth (CMC: 15.0 g of CarboxyMethyl-Cellulose, 1.0 g NH_4_NO_3_, 1.0 g KH_2_PO_4_ monobasic, 0.5 g MgSO_4_7H_2_O, 1.0 g yeast extract, 1 L distilled H_2_O; Tuite, 1969). Flasks were swirled at 90 rpm for 7–9 days under continuous fluorescent light at room temperature. The macroconidial concentration for each isolate was adjusted to 1 × 10^6^ in distilled H_2_O using a hemacytometer. The macroconidial suspension from each isolate was combined in equal proportions.

Common bean genotypes from the MDP (280) and ADP (270) were evaluated for resistance to *F. solani* under greenhouse conditions. For each genotype, three seeds were planted in a single 4-inch plastic pot with drainage holes containing general-purpose PRO-MIX BX General Purpose (Quakertown, PA, United States) potting soil using a randomized complete block design with three replicates (1 replicate = 1 pot). Soil was saturated with water once daily. Inoculations were conducted when hypocotyl arches broke the soil surface by pipetting 5 mL of *F. solani* macroconidial suspension directly to the base of the seedling. Because the genotypes evaluated vary greatly in root architecture and color, a non-inoculated control, one pot containing three seeds, was included as a reference for disease severity ratings for each genotype. Soil was not watered again until plants reached the 80% wilting point by weight. Pots were watered every 2–3 days thereafter to maintain the soil at an 80% wilting point. All plants were maintained in a greenhouse under 16 h of light at 25°C ± 2°C with 90% relative humidity.

To measure assay reproducibility, the ADP was screened in two sub-groups each consisting of 135 lines and the MDP was divided into two sub-groups consisting of 140 lines (Supplementary Tables S1, S2). Susceptible (Montcalm and Cabernet), moderate (Dynasty and Talon), and resistant (VAX3) control lines were included when screening genotypes from each sub-group of the ADP and MDP. These lines were selected based on previously published data and preliminary trials (Bilgi et al., 2008; Vasquez-Guzman, 2016). Therefore, each control line was screened four times and each genotype was screened twice.

### Fusarium Root Rot Evaluation and Data Analyses

Two weeks after inoculation, plants were harvested and roots were washed and evaluated for disease using a 1–9 disease rating scale; 1 = no visible disease symptoms, 3 = light discoloration without necrotic lesions or 10% of the hypocotyl/root tissues covered in root lesions, 5 = approximately 25% of the hypocotyl/root tissue is covered with lesions but the tissue remains firm, 7 = approximately 50% of the hypocotyl/root tissue is covered with lesions with considerable softening and rotting, 9 = approximately 75% or more of the hypocotyl/root tissue is affected with advanced stages of rotting along with significant reduction in root system (Van Schoonhoven and Pastor-Corrales, 1987). Infection in the control lines in each experiment was confirmed to be *F. solani* by isolating the fungus from roots. Roots were surface sterilized in a 0.8% NaOCl solution for 30 s and placed onto 0.5 × potato dextrose agar amended with streptomycin and neomycin, both at a concentration of 50 mg/L. Cultures were morphologically identified to species 1 week following hyphal tipping onto 0.5 × potato dextrose agar (Leslie and Summerell, 2006). The translation elongation factor alpha 1 (TEF-1α) was sequenced as described above to verify morphological identification (Knutsen et al., 2004).

Fusarium root rot severity from the control lines was utilized to evaluate assay reproducibility (Oladzad et al., 2019b). Mean, standard error (SE) of the mean, and coefficients of variability (SE of the mean/mean) were calculated from root rot scores. A one-way ANOVA (α = 0.05) was conducted across the four MDP and ADP sub-group evaluations for each control line (Wong and Wilcox, 2000). Estimated relative treatment effects (ranging from 0 to 1), confidence intervals, and *P-*values were used to determine statistical differences across control lines within each sub-group evaluation (Shah and Madden, 2004; Oladzad et al., 2019b). Significant differences from control lines VAX3, Talon, and Montcalm in MDP and ADP genotypes were based on *P*-values generated from relative effects and associated 95% confidence intervals were calculated using the LD_CI macro in SAS (Domhof and Langer, 2002; Shah and Madden, 2004). Genotypes with relative effects not significantly different from VAX3 were classified as resistant to FRR.

### Genome Wide Association Analysis and Candidate Genes Analysis

Two sets of approximately 200 k imputed SNPs for each diversity panel generated from genotype-by-sequencing (GBS) reads of 325 ADP and 469 MDP genotypes were used for association mapping (Oladzad et al., 2019a). The SNPs were filtered for minor allele frequency ≥ 5% for GWAS analysis. Initially the original data was used from the 1 to 9 scoring system as described earlier. However, different disease classifications identify different genetic factors associated with the resistance response (Oladzad et al., 2019a). Therefore, the disease score data was also evaluated as a binary distribution (score < 3 as resistant and score > 3 as susceptible), and as a three-class polynomial distribution (score < 2.5 as resistant, score = 2.5–3.5 as moderate, and score > 3.5 as susceptible). GEMMA was used for the GWAS analysis because the algorithms programmed in GEMMA can model different types of data distributions (Zhou and Stephens, 2012). For each run, random and mixed models were tested. A kinship matrix generated from the centered relatedness procedure in GEMMA was used as a random effect variable in the random model. A structure matrix generated from principle component analysis (PCA) using Prcom function in R (Price et al., 2006) was used as a fixed effect and together with kinship matrix were tested for a mixed model. Three and four PCAs were employed in model analyses for the ADP and MDP, respectively, accounting for 25–50% variation in each gene pool. *P-wald* test (the improved calibrated *P*-value in GEMMA) was calculated for the given model. The bootstrap distributions of *P*-values were estimated based on 10,000 resamples to determine the significance cutoff at the 0.01 and 0.1 frequency. The best-fitting model was chosen for each of the three phenotypic distributions based on the mean of the squared differences (MSD; Mamidi et al., 2011). The mhtplot function from R package gap was used to create Manhattan and QQ-plots (Zhao, 2007). To estimate the amount of phenotypic variation explained by significant SNPs/regions, a likelihood-ratio-based (R2LR) was calculated using GenABEL package in R (Sun et al., 2010). Finally, the candidate genes were identified based on the best hit on *Arabidopsis thaliana* within a ±50 kb window of the significant SNPs or interval.

## Results

### Fusarium Root Rot Greenhouse Assay Reproducibility

**FIGURE 1:**
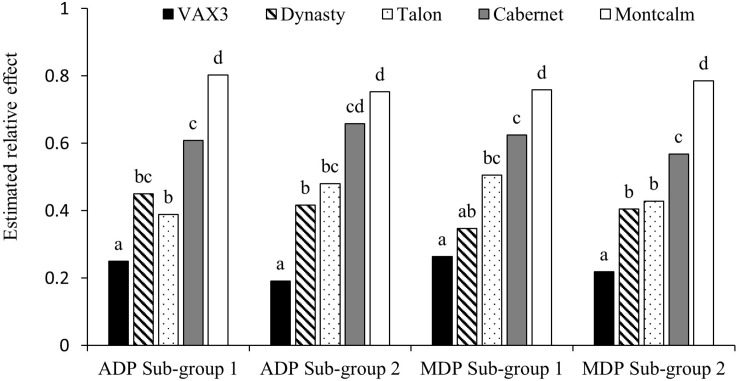
Assay reproducibility of the five control lines when screened for root rot caused by *Fusarium solani* within sub-group evaluations of *Phaseolus vulgaris* Andean Diversity Panel (ADP) and Middle American Diversity Panel (MDP).

Mean disease severity (MDS) for the control lines across the two sub-group evaluations within each panel were not significantly different (Table 1). VAX3 was significantly more resistant to *F. solani* than the other control lines, with the exception of Dynasty in one of four sub-group evaluations (Figure 1). Montcalm was significantly more susceptible to FRR than all other control lines, except for Cabernet in one evaluation. Cabernet was significantly more susceptible than Talon and Dynasty for two and three of four sub-group evaluations, respectively. No significant difference in FRR was observed between Talon and Dynasty across all sub-group evaluations.

**TABLE 1:** Fusarium root rot (FRR) mean disease severity (MDS) across two sub-group evaluations for the Andean Diversity Panel (ADP) and Middle American Diversity Panel (MDP).

Control line	Reaction to FRR^a^	ADP sub-groups		MDP sub-groups	
		MDS^b^	*p*-value^c^	MDS^b^	*p*-value^c^
VAX3	Resistant	1.5	0.45	1.3	1.00
Talon	Moderate	2.5	0.16	2.3	0.90
Dynasty	Moderate	2.7	0.29	2.3	0.52
Cabernet	Susceptible	3.2	0.63	3.8	0.20
Montcalm	Susceptible	4.5	0.58	4.5	0.45

*^a^Reaction to FRR based on previous literature (Vasquez-Guzman, 2016) and preliminary studies. ^b^MDS based on a 1 to 9 scale (Van Schoonhoven and Pastor-Corrales, 1987). ^c^One way ANOVA (α < 0.05) comparing MDS for each control line within diversity panel across sub-group evaluations.*

### Fusarium Root Rot Resistant Genotypes

**FIGURE 2:**
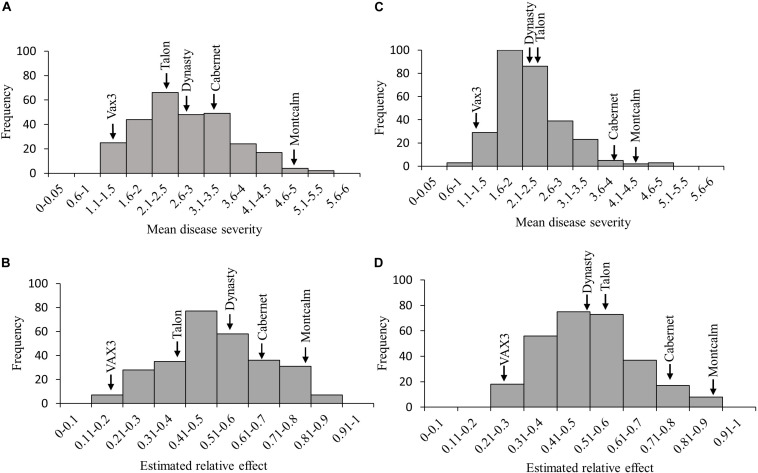
Frequency distribution of **(A)** mean disease severity (MDS) and **(B)** the estimated relative effects of root rot caused by *Fusarium solani* in the Andean Diversity Panel (ADP). Frequency of **(C)** average disease score and **(D)** the estimated relative effects of *Fusarium solani* in the Middle American Diversity Panel (MDP). Arrows indicate the reactions of the five control lines.

Relative effects generated from the FRR ratings for the ADP and MDP resulted in normal distributions (Figure 2). Root rot severity from single plants of genotypes in both the ADP and MDP ranged from 1 to 9. The MDS for the ADP was 2.7 with a range of 1.1–5.4 (Figure 2A). The mean relative effect for the ADP was 0.49 and the range was from 0.18 to 0.86 (Figure 2B). The MDS for the MDP was 2.3 with a range of 1.0–4.8 (Figure 2C). The relative effects for the MDP ranged from 0.24 to 0.87 (Figure 2D) with a mean of 0.50. The estimated relative effects for 34 genotypes from the ADP were not significantly different from the resistant control VAX3 (Table 2 and Supplementary Table S1). Among these 34 were 10 kidney and seven yellow-seeded genotypes. Among the 47 MDP genotypes classified as resistant to FRR were nine from the pinto market class, 11 black, and 10 navy (Table 3 and Supplementary Table S2). All lines from both panels statistically similar to the resistant control VAX3 were significantly different from the susceptible control Montcalm (Supplementary Tables S1, S2). Nine ADP lines were statistically similar to both VAX3 and Talon. All MDP lines displaying root rot statistically similar to VAX3 displayed significantly less root rot than Talon.

**TABLE 2:** Common bean lines from the Andean Diversity Panel (ADP) classified as resistant when compared to the resistant control VAX3 based on overlapping confidence intervals and *P-*values (α < 0.05)^a^.

Line^b^	Marketclass/seed color	Genotype^c^	Mean rank	Est. relative effect^d^	Confidence interval (95%) for relative effect^e^	
					Lower limit	Upper limit
VAX3	Resistant Control	VAX3	52.8	0.19	0.18	0.20
ADP626	Light Red Kidney	Badillo	73.3	0.26	0.14	0.42
ADP640	White Kidney	Beluga	82.0	0.30	0.17	0.46
ADP43	Dark Red Kidney	BWANA_SHAMBA	83.5	0.30	0.17	0.46
ADP99	Dark Red Kidney	BwanaShamba	83.5	0.30	0.17	0.46
ADP511	Yellow	Canario	51.2	0.19	0.18	0.20
ADP513	Yellow	Canario	72.1	0.26	0.18	0.36
ADP186	Red	G1368	52.8	0.19	0.18	0.20
ADP214	Black	G5087	72.3	0.26	0.14	0.42
ADP444	Red Mottled	HondoValle25	72.3	0.26	0.14	0.42
ADP612	Dark Red Kidney	ICA Quimbaya	62.6	0.22	0.16	0.30
ADP683	Pink Mottled	IJR	62.6	0.22	0.16	0.30
ADP621	Yellow	JaloEEP558	73.3	0.26	0.14	0.42
ADP88	Purple Speckled	KABLANKETI	52.8	0.19	0.18	0.20
ADP81	Purple Speckled	KABLANKETI	83.5	0.30	0.17	0.46
ADP519	Sugar	Katarina Cela	72.7	0.26	0.14	0.42
ADP4	Full	KILOMBERO	62.3	0.22	0.16	0.30
ADP94	Yellow	LUSHALA	72.3	0.26	0.14	0.42
ADP684	Dark Red Kidney	Majesty	82.6	0.30	0.17	0.46
ADP514	Yellow	MantegaAmarela	52.8	0.19	0.18	0.20
ADP21	Yellow	MBULAMTWE	81.4	0.30	0.17	0.46
ADP42	Dark Red Kidney	MKOKOLA	73.3	0.26	0.14	0.42
ADP391	Light Red Kidney	PI308894	82.9	0.30	0.17	0.46
ADP392	Sugar	PI309701	72.3	0.26	0.14	0.42
ADP481	Red Mottled	PI449428	62.3	0.22	0.16	0.30
ADP474	Red Mottled	PI527519	72.3	0.26	0.14	0.42
ADP462	Yellow	PI527540B	83.9	0.30	0.17	0.46
ADP429	Pink Cranberry	PR9920_171	72.7	0.26	0.14	0.42
ADP1	Red Mottled	ROZI_KOKO	52.8	0.19	0.18	0.20
ADP602	Light Red Kidney	Sacramento	78.7	0.27	0.14	0.47
ADP112	Red	Uyole96	71.6	0.26	0.14	0.42
ADP111	Sugar	Uyole98	52.8	0.19	0.18	0.20
ADP2	Purple Speckled	W6_16444	72.9	0.26	0.14	0.42
ADP15	Dark Red Kidney	W6_16495	71.6	0.26	0.14	0.42
ADP91	Manteca	W6_16560	73.3	0.26	0.14	0.42

*^a^P-values (α = 0.05) were generated as previously described (Altman and Bland, 2011). ^b^ADP as previously described (Cichy et al., 2015). ^c^VAX3 was utilized as the resistant control. ^d^Estimated relative effect was determined using the mean rank for each bean line among all the observations within the experiment. Relative effects range between the values of 0 and 1.0 (Shah and Madden, 2004). ^e^95% confidence intervals were generated from the estimated relative effect values.*

**TABLE 3:** Common bean lines from the Middle American Diversity Panel (MDP) classified as resistant as compared to the resistant control VAX3 based on overlapping confidence intervals and *P-*values (α < 0.05)^a^.

Line^b^	Marketclass/seed color	Genotype^c^	Mean rank	Est. relative effect^d^	Confidence interval (95%) for relative effect^e^	
					Lower limit	Upper limit
VAX3	Resistant Control	VAX3	65.9	0.24	0.23	0.26
MDP113	Pinto	Fargo	65.9	0.24	0.23	0.26
MDP126	Black	Loreto	71.2	0.24	0.23	0.26
MDP134	Navy	Navigator	65.8	0.24	0.23	0.26
MDP140	Small Red	Ember	65.9	0.24	0.23	0.26
MDP216	Black	I9365_31	65.9	0.24	0.23	0.26
MDP267	Pink	Victor	65.9	0.24	0.23	0.26
MDP332	Black	CDC_Jet	65.9	0.24	0.23	0.26
MDP349	Black	Harrowhawk	65.9	0.24	0.23	0.26
MDP52	Pinto	I06_2575_17	65.9	0.24	0.23	0.26
MDP55	Navy	Sanilac	67.3	0.24	0.23	0.26
MDP9	Small Red	AC_Redbond	65.9	0.24	0.23	0.26
MDP142	Pink	ROG_312	79.5	0.30	0.21	0.41
MDP167	Pinto	UI_126	78.8	0.30	0.21	0.41
MDP302	Pinto	ND_307	75.5	0.30	0.21	0.41
MDP32	Black	DPC_4	78.8	0.30	0.21	0.41
MDP329	Great Northern	CDC_Crocus	78.5	0.30	0.21	0.41
MDP331	Black	CDC_Expresso	80.9	0.30	0.21	0.41
MDP403	Navy	McHale	78.5	0.30	0.21	0.41
MDP129	Navy	Voyager	80.7	0.31	0.20	0.44
MDP131	Pink	Pink_Floyd	79.8	0.31	0.20	0.44
MDP187	Great Northern	GN_Star	82.9	0.32	0.19	0.49
MDP290	Tan	BAT_477	85.3	0.32	0.19	0.49
MDP13	Navy	AC_Polaris	88.8	0.33	0.19	0.51
MDP14	Great Northern	AC_Resolute	88.0	0.33	0.19	0.51
MDP146	Black	Black_Knight	87.2	0.33	0.19	0.51
MDP15	Small Red	AC_Earlired	88.9	0.33	0.19	0.51
MDP159	Small Red	UI_37	88.9	0.33	0.19	0.51
MDP201	Great Northern	NE1_09_20	89.1	0.33	0.19	0.51
MDP203	Pinto	NE2_09_1	88.8	0.33	0.19	0.51
MDP239	Pinto	USPT_CBB_5	89.1	0.33	0.19	0.51
MDP268	Pink	USWA_61	88.8	0.33	0.19	0.51
MDP286	Pink	A285	88.9	0.33	0.19	0.51
MDP3	Pinto	BelDakMi_RR_5	88.8	0.33	0.19	0.51
MDP383	Pinto	Apache	87.2	0.33	0.19	0.51
MDP395	Black	Black_Velvet	88.0	0.33	0.19	0.51
MDP43	Small Red	TARS09_RR007	88.0	0.33	0.19	0.51
MDP61	Navy	Neptune	87.2	0.33	0.19	0.51
MDP66	Black	C_20	87.2	0.33	0.19	0.51
MDP7	Great Northern	BelNeb_RR_1	89.1	0.33	0.19	0.51
MDP78	Navy	Mackinac	89.1	0.33	0.19	0.51
MDP96	Black	Cornell 49-242	88.8	0.33	0.19	0.51
MDP99	Pink	S08418	87.2	0.33	0.19	0.51
MDP133	Navy	Medalist	93.8	0.35	0.18	0.56
MDP339	Navy	Nautica	92.9	0.35	0.18	0.56
MDP382	Pinto	Sequoia	92.9	0.35	0.18	0.56
MDP392	Black	B05055	96.0	0.35	0.18	0.56
MDP90	Navy	Albion	91.3	0.35	0.18	0.56

*^a^P-values (α = 0.05) were generated as previously described (Altman and Bland, 2011). ^b^MDP as previously described (Moghaddam et al., 2016). ^c^VAX3 was utilized as the resistant control. ^d^Estimated relative effect was determined using the mean ranks for each bean line among all the observations within the experiment. Relative effects range between the values of 0 and 1.0 (Shah and Madden, 2004). ^e^95% confidence intervals were generated from the estimated relative effect values.*

### Genome Wide Association Analysis and Candidate Genes in the ADP

**FIGURE 3:**
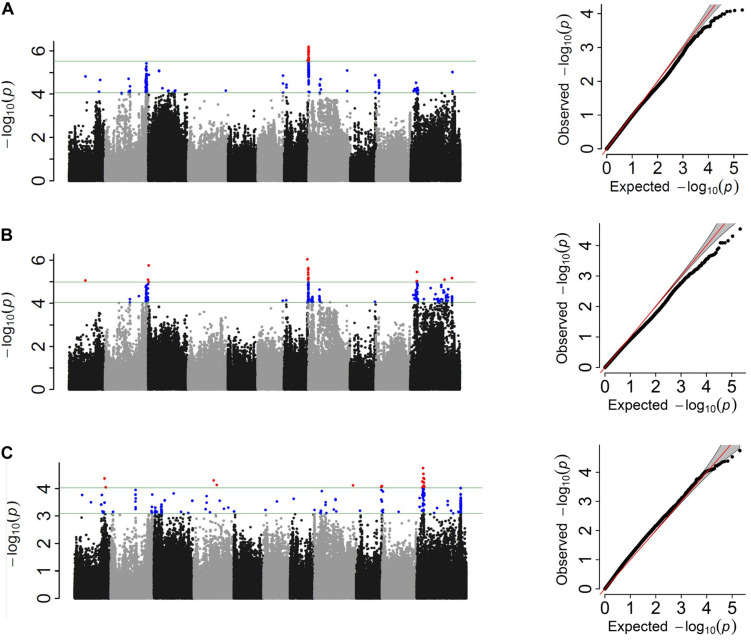
Manhattan and corresponding Q–Q plots representing the genetic architecture of *Fusarium solani* resistance from GWAS analysis of **(A)** quantitative, **(B)** three-class, and **(C)** binary scoring systems in the Andean Diversity Panel (ADP).

HapMap SNPs totaled 260,670 and 205,293 for the ADP and MDP, respectively^1^. After filtering for MAF, 219,056 SNPs for ADP and 125,745 SNPs for MDP were used for association study. Three GWAS analyses based on different phenotypic distributions detected common and unique SNPs or intervals associated with FRR. Two common genomic regions (Pv08:0.04–0.18 Mbp and Pv07:38.5 Mbp) were associated in the ADP in both the score and three-class phenotyping system at the *P* < 0.01 significance level (Figure 3). These two regions cumulatively explained 17 and 19% of the phenotypic variation in score and three-class analyses, respectively. However, a large significant genomic interval on Pv11:9.10–9.47 was detected in three-class (11% phenotypic variation explained) and binary (14%) analyses but not in score analysis. Unique SNPs at this threshold were also identified independently in each analysis (Table 4). Overall, binary data explained the most cumulative phenotypic variation (37%) and the significant SNPs detected in three-class data explained the most individual phenotypic variation associated with FRR in the ADP. When looking at the less stringent criteria (0.1% cutoff level), 151 SNPs were discovered to be common at least between two phenotyping system (Supplementary Table S3). From this, 17 SNPs were identified in all three systems. Fourteen SNPs were common between the score and binary analyses, 93 SNPs were common between score and three-class analyses, and 27 SNPs were common between binary and three-class analyses. Most of the SNPs on Pv11 were detected in binary and three-class analyses, while most of the SNPs on Pv02 and Pv08 were detected in score and three-class. When comparing the three phenotypic scoring analyses in the ADP GWAS analysis, the three-class scoring had the most frequent SNPs in common with another scoring system.

**TABLE 4:** Significant genomic regions/SNPs from GWAS in Andean Diversity Panel (*P* < 0.01).

Phenotypic data	Interval		Peak SNP		*R* square	Cumulative R square
	Chrom	Genomic interval (Mb)	Position (Mb)	−Log10(P)		
Score	8	0.04-0.18	S08_184683	6.18	0.16	0.17
	7	38.5	S07_38504717	5.53	0.14	
Three class	1	26.86	S01_26860673	5.05	0.12	0.32
	2	49.43	S02_49439131	5.01	0.13	
	3	1.07	S03_1079664	5.75	0.15	
	7	38.5	S07_38504717	6.04	0.15	
	8	0.04–0.18	S08_184683	5.64	0.14	
	11	9.10–9.47	S11_9423668	5.46	0.14	
	11	37.06	S11_9472666	5.03	0.12	
	11	44.79	S11_44799085	5.16	0.13	
Binary	11	8.13	S11_8136772	4.25	0.10	0.37
	11	9.10–9.47	S11_9106599	4.7	0.11	
	11	10.01–10.04	S11_10397908	4.34	0.09	
	1	42.16	S01_42160134	4.37	0.10	
	1	44.34	S01_44346150	4.03	0.08	
	4	24.87	S04_24875044	4.29	0.09	
	4	28.34	S04_28344173	4.12	0.09	
	8	60.07	S08_60070729	4.11	0.09	
	9	38.06	S09_38068347	4.07	0.09	

Candidate genes were searched for the peak SNPs at the 0.01 cutoff level and 52 potential candidate genes were identified from all three analyses (Supplementary Table S4). Twenty bean gene models were associated with genomic regions Pv08:0.04–0.18 Mbp, and 13 were previously characterized in Arabidopsis. The peak SNP at this interval was located inside the gene model *Phvul.008G001300* which encodes an ortholog of the glucan synthase-like enzyme (*GSL*) associated with the deposition of callose in papillae at pathogen wound sites (Ellinger and Voigt, 2014). A second gene, *Phvul.008G002250*, located 59 kb down stream of this peak SNP, also encodes a glucan synthase-like protein. Two other gene models, *Phvul.008G001100* and *Phvul.008G001200*, both orthologs of senescence-associated genes (*SAG*) involved in disease defense in plants, were also located within this interval. Both *SAG* and *GSL* genes clustered with the NAC domain protein *Phvul.008G001000*, which is also associated with fungal disease (Hickman et al., 2013). The SNP peak at Pv07:38.5 Mbp was located inside Homogentisate prenyltransferase gene encoding for plasoquinon. Eighteen common bean gene models were associated with Pv11:9.10–9.47 genomic interval. *Phvul.011G092600* maps within this interval, a member of the Subtilisin-like serine endopeptidase family protein that is involved in plant-pathogen interactions (Figueiredo et al., 2014). Additionally, eight candidate genes unique to the binary analysis and six candidate genes unique to the three-class analysis were associated with FRR. *Phvul.002G329400*, an ortholog of the pathogenesis-related thaumatin, and *Phvul.003G009700*, a member of the pentatricopeptide repeat (*PPR*) superfamily, were located in significant regions. Presumed functions of these are related to the plant pathogen resistance response (Geddy and Brown, 2007). WRKY DNA-binding protein, P-loop containing nucleoside triphosphate hydrolases, and Leucine-rich receptor-like protein kinase family proteins associated with gene models *Phvul.008G251700*, *Phvul.004G086200*, and *Phvul.009G260500* in binary analysis are also noted for their role in plant disease resistance (Ellis et al., 2000; Yu et al., 2001).

### Genome Wide Association Analysis and Candidate Genes in the MDP

**FIGURE 4:**
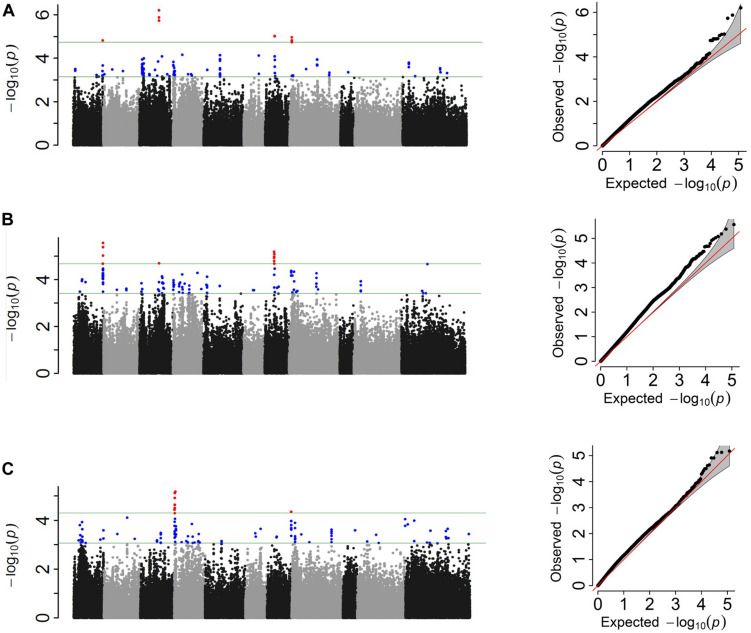
Manhattan and corresponding Q–Q plots representing the genetic architecture of *Fusarium solani* resistance from GWAS analyses using **(A)** quantitative, **(B)** three-class, and **(C)** binary scoring systems in Middle American Diversity Panel (MDP).

The Pv01:51.03–51.07 Mbp and Pv07:15.29 intervals were shared between the score and three-class analyses at the 0.01 cutoff (Figure 4 and Table 5). These intervals explained 8 and 9% of the phenotypic variation in score and three-class analyses, respectively. The binary analysis did not share any significant SNP at this significance level with the other two phenotyping analyses. The largest cumulative phenotypic effect, 27%, was observed for the significant SNPs when using phenotypic score. Significant regions on Pv04 were detected when using the binary analysis. However, more shared SNPs were detected among all three analyses when SNPs significant at the 0.1 cutoff were considered (Supplementary Table S5). A total of 51 significant SNPs were shared between at least two scoring systems, of which eight SNPs were shared among all three, six SNPs were shared between score and binary systems, 29 SNPs were shared between score and three-class systems, and eight SNPs were shared between binary and three-class. In addition to the shared SNPs, each analysis discovered unique SNPs associated with FRR (Supplementary Table S5).

**TABLE 5:** Significant genomic regions/SNPs from GWAS in Middle American Diversity Panel (*P* < 0.01).

Phenotypic data	Interval		Peak SNP		*R* square	Cumulative R square
	Chrom	Genomic interval (Mb)	Position (Mb)	−Log10(P)		
Score	3	25.26	S03_25262205	6.2	0.10	0.27
	7	15.29	S07_15295231	5.02	0.08	
	8	5.10-5.11	S08_5112094	4.9	0.08	
	1	51.03	S01_51036396	4.82	0.07	
Three class	1	50.79-50.82	S01_50822097	4.68	0.07	0.20
	1	51.03-51.07	S01_51036396	5.56	0.09	
	7	15.29	S07_15295263	5.18	0.08	
Binary	4	0.36-0.43	S04_376163	5.12	0.08	0.09
	4	1.31	S04_1315429	5.17	0.08	

Candidate genes were searched for the peak SNPs at the 0.01 cutoff level and 27 potential candidate genes were identified across all three analyses (Supplementary Table S6). Ten gene models were associated with the genomic interval Pv01:51.03–51.07 Mbp. Peak SNP Pv01:51.03 Mbp was located 2 kb upstream of gene model *Phvul.001G263600*, an ortholog of 15-*cis*-zeta-carotene isomerase (*Z-ISO*). Two gene models, *Phvul.001G263700* and *Phvul.001G263800*, orthologs of Immunoglobulin E-set superfamily protein and MAC/Perforin domain-containing protein respectively, were identified upstream of this peak SNP and were detected only in the three-class analysis. Both genes are involved in plant disease defense (Morita-Yamamuro et al., 2005; Zhang et al., 2014). The SNP peak Pv07:15.2 Mbp was located 10kb upstream of gene model *Phvul.007G127800*, an ortholog of ethylene response factor 1 (*ERF*) which regulates plant resistance to some soil-born fungi (Berrocal-Lobo and Molina, 2004). A cluster of cytochrome P450/family 96/subfamily A/polypeptide 10 (*CYP96A10*) was associated with Pv04:37.0–40.34 Mbp in binary analysis. Finally, gene models *Phvul.003G098500* and *Phvul.008G057500*, orthologs of DUF679 domain membrane protein (*DMP*) and GRIP-related ARF-binding domain-containing protein (*GDAP*) respectively were associated with Pv03:25.26 Mbp, Pv08:5.10–5.11 Mbp when using the score system analysis.

## Discussion

This research provides a reproducible greenhouse assay for the evaluation of resistance to FRR. Greenhouse assays were demonstrated as reproducible with the consistent results from five control lines. The inclusion of three of these control lines is recommended for continuing FRR evaluations and ongoing comparisons across research studies. VAX3 and Montcalm have been previously documented as appropriate resistant and susceptible controls, respectively (Bilgi et al., 2008; Vasquez-Guzman, 2016). Those observations were consistent with our results; therefore, we recommend the inclusion of these two lines in future FRR evaluations. Additionally, VAX3 and Montcalm were resistant and susceptible to Rhizoctonia root rot, respectively, making them excellent choices for control lines to be included in field studies where both *Fusarium* spp. and *Rhizoctonia solani* Kühn (teleomorph *Thanatephorus cucumeris*) are likely playing a role in the root rot complex (Oladzad et al., 2019b). In addition to resistant and susceptible controls, the inclusion of a line consistently displaying a moderate reaction to a quantitative trait like root rot resistance is important to fully characterize lines evaluated in all studies. Talon, Dynasty, and Cabernet generally all displayed a moderate reaction to FRR in the current evaluations. FRR severity of these three lines was significantly different from both VAX3 and Montcalm with only one exception; therefore, any of these would be appropriate for inclusion in future studies.

It is particularly difficult to generate reproducible results when evaluating quantitatively inherited traits. Environmental parameters, microbial interactions, and screening methods present numerous opportunities for data inconsistencies. These variables are exacerbated when handling a soil-borne pathogen complex. Soil-borne plant pathogens exist simultaneously with a variety of non-pathogenic soil microorganisms, creating complexes, and therefore, the focus of research concerning soil-borne plant pathogens is being redirected to considering complexes rather than individual strains of individual species (Lamichhane and Venturi, 2015; Abdullah et al., 2017). Similar to other *Fusarium* species complexes, species within the FSSC display varying degrees of aggressiveness (Chitrampalam and Nelson, 2016). Therefore, the approach taken here to incorporate multiple isolates/strains from the FSSC in phenotypic evaluations is more representative of field conditions and contributed to assay reproducibility.

Successful infection by a soil-borne pathogen is heavily dictated by environmental parameters. Controlling or predicting the soil environment is complicated; therefore, determining host resistance against soil-borne pathogens may not be applicable across environments. Of the numerous soil parameters that dictate the infection rate and severity of FRR pathogens, the effects of soil temperature and moisture have been most documented (Porch et al., 2014; Teixeira et al., 2015; Macedo et al., 2017). In North Dakota, variability in growth rates and aggressiveness were observed across a range of temperatures for 96 species in the FSSC (Chitrampalam and Nelson, 2016). Most *Fusarium* spp. require some type of plant stress to incite infection on pulse crops; such stress may include droughts or flooding (Leslie et al., 1990). Soil moisture also has been documented to influence the presence and inoculum density of FSSC (Macedo et al., 2017). Flooding and drought conditions have been reported to substantially weaken the dry bean root system and allow infection caused by FRR (Gossen et al., 2016; Macedo et al., 2017). To date, this is the first FRR screening method to account for soil moisture and the microbial interactions of numerous strains within the FSSC, likely contributing to assay reproducibility.

While field trials are the truest test of pathogen resistance, they are laden with many challenges. Soil-borne pathogens are rarely evenly distributed throughout naturally infested fields, contributing to potentially inconsistent results, particularly when screening a large number of lines. In addition to affecting host susceptibility, the level of disease pressure is greatly affected by environmental conditions. Field trials are constantly threatened by weather events including hail, flooding, or drought. In contrast, greenhouse evaluations somewhat ignore interactions across the soil microbiome and soil environmental factors that affect disease development. However, greenhouse screening can provide the opportunity for consistently identifying genotypes with resistance to a single pathogen over a short time period, as is demonstrated here. The FRR resistance data presented here is more dynamic due to the implementation of a new greenhouse screening method utilizing a mixture of isolates and drought stress to promote disease development on a large set of genotypes (ADP = 270; MDP = 280). Thirty-four ADP and 47 MDP resistant genotypes were identified from the most important market classes in both the Middle American and Andean gene pools including pinto, black, great northern, and dark red kidney. The identification of these genotypes will provide breeding programs with valuable germplasm for incorporation of resistance to this important soil-borne pathogen.

Previous research for screening of FRR resistance either utilized single isolates of *F. solani* or were conducted in naturally pathogen-infested fields (Román-Avilés and Kelly, 2005; Bilgi et al., 2008; Nicoli et al., 2012; Conner et al., 2014; Hagerty et al., 2015; Nakedde et al., 2016; Vasquez-Guzman, 2016). However, the FRR reaction of some genotypes evaluated in this study were supported by results from previous research. The ADP genotype PI5275408B was characterized as resistant, Etna, Fox Fire, Pink Panther, W6_6534, and 46_1 were characterized as moderately resistant, and Montcalm were characterized as susceptible (Bilgi et al., 2008; Conner et al., 2014; Vasquez-Guzman, 2016). Similarly, some previously screened MDP genotypes, AC_Polaris, AC_Resolute, and AC_Earlired (resistant), Navigator, CDC_Jet, AC_Redbond, AC_Island, Black Violetand Zorro (moderately resistant), and Beryl, Envoy, Matterhorn, and Othello (susceptible) were confirmed with same response in our study (Bilgi et al., 2008; Conner et al., 2014; Nakedde et al., 2016).

Some of the FRR resistant genotypes identified in this study also displayed resistance to Rhizoctonia root rot. ADP genotypes ROZI_KOKO, W6_16495, and HondoValle25 and MDP genotypes USWA_61, Nautica, B05055 previously described as resistant to *R. solani* AG2-2 are also resistant to FRR in the present study (Oladzad et al., 2019b). These genotypes provide a unique opportunity for the incorporation of resistance to at least two important soil-borne pathogens of the common bean. Two genomic regions on Pv02 and PV11 identified in this study were also associated *R. solani* resistance in ADP panel (Oladzad et al., 2019b). However, further investigation is needed to determine if the resistance to these two pathogens is due to root architecture or some feature other than host resistance. In a previous study, FRR was determined to be controlled by root genotype and root vigor played an important part in resistance (Cichy et al., 2007). Wang et al. (2018) demonstrated that resistant lines had a slightly higher root biomass and hypothesized that some of the QTL associated with FRR resistance are more likely related to root biomass. High resistance consistently associated with root architecture, which indicates that these may be dependent traits and need to be considered when selecting lines for resistance breeding (Strock et al., 2019).

This study is the first to utilize ∼200 K SNPs to identify SNPs closely associated with FRR resistance in major common bean gene pools. These high-quality SNPs obtained through GWAS in this study will provide the foundation for confident subsequent analyses of candidate genes and can be converted into breeder friendly markers to aid in the incorporation of FRR resistance in high yielding lines through marker assisted selection. In the current study, the genomic regions associated with response to *F. solani* in the common bean were identified independently in two diversity panels representing modern germplasm from the two common bean gene pools. As previous studies suggested, different genetic factors might be involved for the same traits in each common bean gene pool (Schmutz et al., 2014; Soltani et al., 2017, 2018; McClean et al., 2018; Oladzad et al., 2019a, b). Previous work on *R. solani* (Oladzad et al., 2019b) demonstrated that shared and/or unique genomic regions were significantly associated with disease severity when the data was considered on a 1–9 scale, or transformed into binary or multinomial distributions. For this reason, three independent GWAS analyses were performed using the score data and three-class and binary phenotypic data for each diversity panel. As expected, each GWAS analysis detected SNPs specific to each phenotypic data set as well as shared significant regions. Identifying unique, as well as shared, SNPs using three phenotypic approaches is consistent with GWAS results previously observed for *R. solani* resistance in the common bean (Oladzad et al., 2019b). In general, for both the ADP and MDP, genomic intervals discovered with the three-class phenotypic distribution data were shared with results obtained GWAS results for the binary and the 1–9 distribution data. However, in searching for candidate genes, it was observed that each phenotyping data set used for GWAS analysis offered specific important genetic regions associated with FRR. The ADP GWAS identified 52 potential candidate genes from all three analyses, and of these, nine candidates were previously documented for their roles in plant pathogen response. During evolution, plants developed successful immune/defense mechanisms against pathogen infections. One of these mechanisms was discovered from expression studies on Glucan synthase-like protein (*GSL*), which is a potential ADP candidate gene (*Phvul.008G001300*). *GSL* regulates callose synthesis, which are abundant components of the papillae structure at sites of fungal penetration (Voigt, 2014). The formation of this complex structure appears at the earliest phase of the plant defense response to pathogen infection. It has been shown in Arabidopsis that the elevated cell wall callose polymers in papillae provides complete pathogen penetration resistance (Ellinger et al., 2013). In our study, *GSL* were found in the vicinity of NAC domain and senescence-associated genes (*SAG*). NAC domains generally have important roles in the regulation of both biotic and abiotic stresses in plants. However, significant progress in NAC domain function studies revealed the important role of these domains in activating plant’s defense responses. It has been shown that both positive and negative regulatory roles of NAC transcription factors (TFs) in the alteration of gene expression are the key mechanisms employed by plants during pathogen attack (Nuruzzaman et al., 2013). Most of these genes regulate signaling of plant hormones during the immune response (Yuan et al., 2019). More importantly, *NAC* TFs and *SAG* genes seem to be closely related to the biotic stress response. Pathogen infection is one of the factors that affects the signaling pathway of senescence in plants. NAC subfamily proteins are involved in altering the regulation of senescence signaling pathway (Guo and Gan, 2006), and these *NAC* TFs regulate salicylic acid (*SA*) and jasmonic acid (*JA*) signaling pathways, both known to accelerate developmental senescence in plants (Hickman et al., 2013). Taken together, detecting a cluster of these three genes in the vicinity of each other on Pv08 supports the idea that these are the most important potential candidate genes associated with *F. solani* response in the ADP.

Subtilisin-like serine and WRKY DNA-binding protein (*WRKY*) genes also mapped to significant genomic regions in the ADP on Pv11 and Pv08, respectively. The first evidence of subtilisin-like serine related to plant pathogenesis was reported in tomatoes (Tornero et al., 1996). Subsequently it was shown that, in soybeans, a plant defense peptide signal (*GmSubPep*) is embedded in subtilisin-like protein (*Glyma18g48580*) and upon pathogen attack, this peptide is accessible to activate defense-related genes (Pearce et al., 2010). In grapes, a subgroup of subtilisin-like proteins exhibit slight structural modifications in varieties resistant to *Plasmopara viticola* to affect plant programmed cell death (PCD) at the site of pathogen attack, such that the pathogen is not able to recognize this protein and prevents the PCD response that is a component of the plant immune resistance mechanism (Gindro et al., 2012; Figueiredo et al., 2014). WRKY DNA binding proteins act upstream of pathogenesis-related genes (*PRs*) and positively regulate their expression upon plant infection by a pathogen (Yu et al., 2001). One PR gene model (*Phvul.002G329400*) was detected in the ADP three-class GWAS analysis, which could be the target of this *WRKY* gene (*Phvul.008G251700*).

In MDP GWAS analysis, 27 potential candidate genes from all three GWAS analysis were associated with resistance to FRR. Many are candidate genes previously documented for their roles in plant pathogen response. On Pv04, a significant peak was located in a cluster of *CYP450* genes. These are members of one of the largest protein families in plants, and they are involved in many diverse biological processes, including metabolism pathways and hormonal responses to biotic and abiotic stresses (Bak et al., 2011). This cluster maps adjacent to a *NB-ARC* disease resistance gene cluster. Both clusters might be potential candidate regions associated with FRR. An ethylene response factor 1 (*ERF1*) was associated with peak SNP on Pv07. *ERF1* is a transcriptional factor (TF) with a role in plant resistance to *Fusarium* spp. and necrotrophic fungi (Lorenzo et al., 2003; Berrocal-Lobo and Molina, 2004; van Loon et al., 2006; Van der Ent and Pieterse, 2018). Upon pathogen attack, *ERF1* TFs trigger the activation of *PR* genes through a positive regulation of *JA* gene expression. A Pv01 candidate, a MAC/Perforin domain-containing gene, is part of the perforine membrane attack complex which plays a key role in both plant and animal innate immunity. In Arabidopsis, it has been shown that *MACPF* is encoded by *constitutively activated cell death 1* gene (*CAD1*) that is negatively regulated by the *SA* signaling pathway. Therefore, in resistant plants, the mutant form (*cad1*) activates expression of *PR* genes, leading to *SA* enhancement of the programmed cell death and thus restricting pathogen growth (Morita-Yamamuro et al., 2005; Fukunaga et al., 2017).

Some common SNPs were found to be closely linked between Rhizoctonia and Fusarium root rot resistance in GWAS studies across the MDP and ADP (Oladzad et al., 2019b). The significant genomic region Pv02:49.43 Mb detected in this study is in the vicinity of genomic region Pv02:48.38–49.41 Mb associated with Rhizoctonia resistance in the ADP. Moreover, Pv09:38.06 and Pv11:8.13 Mb associated with Fusarium resistance in this study were near Rhizoctonia resistance genomic region Pv09:31.61 and Pv11:7.84 Mb in the same gene pool. Genomic region detected at Pv01:50.82 Mb was 10.62 Mb upstream and genomic region Pv08:5.11 Mb was 12.74 Mb downstream of the significant genomic regions were reported in Rhizoctonia resistance in the MDP. Therefore, based on our evaluations, some common genetic factors may be involved in the resistance of both Rhizoctonia and Fusarium root rot in the common bean.

To our knowledge, this is the first GWAS study on over 500 genotypes across the main gene pools in the common bean using a large number of SNP markers for studying genetic basis of resistance to FRR. Overall, most of the candidate genes detected in both gene pools seems to be involved in signaling pathways such as *SA* and *JA* through activating the expression of *PR.* However, the significant SNPs detected in each gene pool can be used in common bean breeding to speed up and lower the cost of selecting for resistance to this pathogen.

## Data Availability Statement

The datasets generated for this study can be found in the http://arsftfbean.uprm.edu/beancap/research/.

## Author Contributions

JP, JO, and KZ-A designed and conceived the experiments. KZ-A and CM performed the experiments in the greenhouse. AO, KZ-A, and SJ performed the data analysis and drafted the manuscript. PM and JP supervised the data analysis. JP, KZ-A, SJ, and AO discussed the results and interpretation of the final data. PM, JP, and JO provided suggestions to improve it. All authors read and approved the final manuscript.

## Conflict of Interest

The authors declare that the research was conducted in the absence of any commercial or financial relationships that could be construed as a potential conflict of interest.

## Footnotes

1 http://arsftfbean.uprm.edu/beancap/research/
